# Case report: Exceptional disease progression in a 70-year-old patient: generalized melanosis and melanuria in the course of metastatic melanoma - a case study

**DOI:** 10.3389/fonc.2024.1332362

**Published:** 2024-01-29

**Authors:** Jacek Calik, Piotr Dzięgiel, Natalia Sauer

**Affiliations:** ^1^ Department of Clinical Oncology, Wroclaw Medical University, Wrocław, Poland; ^2^ Old Town Clinic, Wroclaw, Poland; ^3^ Division of Histology and Embryology, Department of Human Morphology and Embryology, Wroclaw Medical University, Wrocław, Poland; ^4^ Department of Human Biology, Faculty of Physiotherapy, Wroclaw University of Health and Sport Sciences, Wrocław, Poland; ^5^ Faculty of Pharmacy, Wroclaw Medical University, Wrocław, Poland

**Keywords:** generalized melanosis, metastatic melanoma, melanuria, BRAF mutation, melanoma

## Abstract

This case study documents an extraordinary disease progression in a 70-year-old patient diagnosed with metastatic melanoma. The patient’s condition advanced to an unusual manifestation characterized by generalized melanosis and melanuria, a rare and foreboding complication of metastatic melanoma. The clinical presentation involved rapid-onset skin darkening, primarily affecting the face and torso, along with darkened urine, marking the onset of melanuria. Despite extensive diagnostic evaluations, including abdominal ultrasound, neck ultrasound, thoracic CT scans, and endoscopic examinations, the exact metastatic sites remained elusive, demonstrating the diagnostic challenges associated with this condition. Laboratory tests revealed abnormal hematological and biochemical markers, along with elevated S100 protein levels, indicating disease progression. The patient underwent a surgical skin biopsy that confirmed the diagnosis of metastatic melanoma, leading to a multidisciplinary approach to treatment. Following this, the patient-initiated chemotherapy with dacarbazine (DTIC). Regrettably, this was necessitated by the absence of reimbursement for BRAF and MEK inhibitors as well as immunotherapy, and it subsequently led to rapid disease progression and a decline in the patient’s clinical condition. The patient’s condition further complicated with erysipelas and increased distress, ultimately leading to their unfortunate demise. This case highlights the aggressive nature of generalized melanosis, characterized by a rapid clinical course, substantial pigmentation, and limited response to conventional chemotherapy. Importantly, the patient had a BRAF mutation, emphasizing the urgency of exploring alternative treatment strategies. Patients with a BRAF mutation are excellent candidates for BRAF and MEK inhibitor treatment, potentially allowing them to extend their lifespan if this therapy were available. The challenges encountered in diagnosing, managing, and treating this aggressive form of metastatic melanoma underline the need for early detection, tailored therapeutic approaches, and ongoing research efforts to improve patient outcomes in such cases.

## Introduction

Worldwide, cutaneous melanoma manifests as a disease with an incidence rate of moderate to low frequency, estimated at approximately 3.8 cases for men and 3.0 cases for women per 100,000 individuals annually. Its primary anatomical location is the trunk (35%) and lower limbs (34%), followed by the upper limbs (20%) and the head and neck (11%) (1). The prevailing subtype among the Caucasian population is Superficial Spreading Melanoma (SSM), with Nodular Melanoma (NM) occupying the second most common position (2). NM typically exhibits a more tumultuous clinical course and frequently arises from previously unaltered skin, in a *de novo* manner (3, 4).

Generalized melanosis represents an infrequent sequel of metastatic melanoma, with a limited number of instances, documented in English medical literature (5). It is regarded as a foreboding indicator, marked by a median survival period of approximately 6 months. This condition is typified by a slate-blue discoloration, frequently intensified in regions exposed to sunlight. Additionally, mucosal surfaces and nail beds manifest signs of involvement. The majority of instances are correlated with the presence of liver metastases (6). Melanuria is a relatively common occurrence in approximately 15% of metastatic melanoma cases (7, 8). It results from the excretion of melanin precursors that oxidize to form melanin in the urine or extracellular melanin granules. This can lead to acute renal injury, although the exact underlying mechanisms remain unclear (9). Potential explanations include the oxidation of melanin precursors released by the tumor, migration of circulating melanophages to the dermis, deposition of melanosomes in the skin, alterations in dermal lymphatics, rare dermal invasion of pigmented single-cell metastases, and increased melanogenesis along with pigment incontinence due to elevated melanocyte peptide growth factors (10–12).

Herein, we present a detailed case report concerning an exceedingly rare pattern of generalized melanosis, affecting both the integumentary system and mucous membranes, along with extracutaneous dissemination to internal organs. Remarkably, instances akin to this presentation are scarcely documented in the English-language medical literature, with a count of approximately 30 such cases. Regrettably, due to the aggressive nature of the disease, most affected individuals were precluded from receiving systemic therapeutic interventions.

## Case presentation

The patient, a 70-year-old individual with Type I skin (characterized by very fair skin, blue eyes, and a propensity to sunburn easily), presented with multiple underlying conditions, including type 2 diabetes, hypertension, and atherosclerosis. The patient had previously undergone angioplasty with stent implantation due to unstable angina pectoris and had also undergone a radical excision of a nodular malignant melanoma on their back, specifically a Clark IV lesion with a Breslow thickness of 3 mm, originating from a pigmented lesion. During the surgical procedure, enlarged lymph nodes in the left axillary region were observed, raising suspicion of metastatic involvement and leading to a lymphadenectomy.

**Figure 1:**
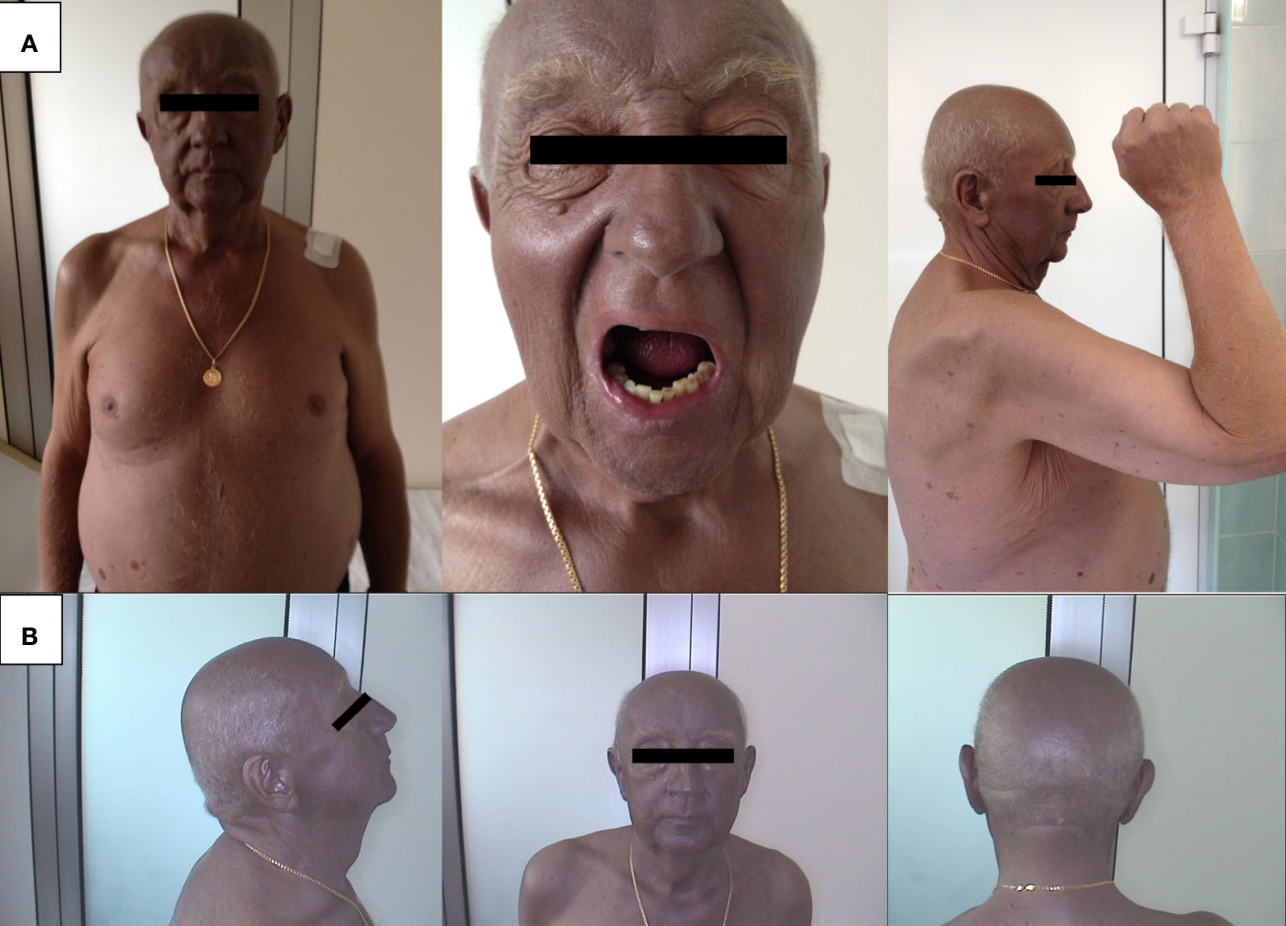
**(A)** Dark skin pigmentation.; **(B)** Progressive dark pigmentation of the skin over time.

**Figure 2:**
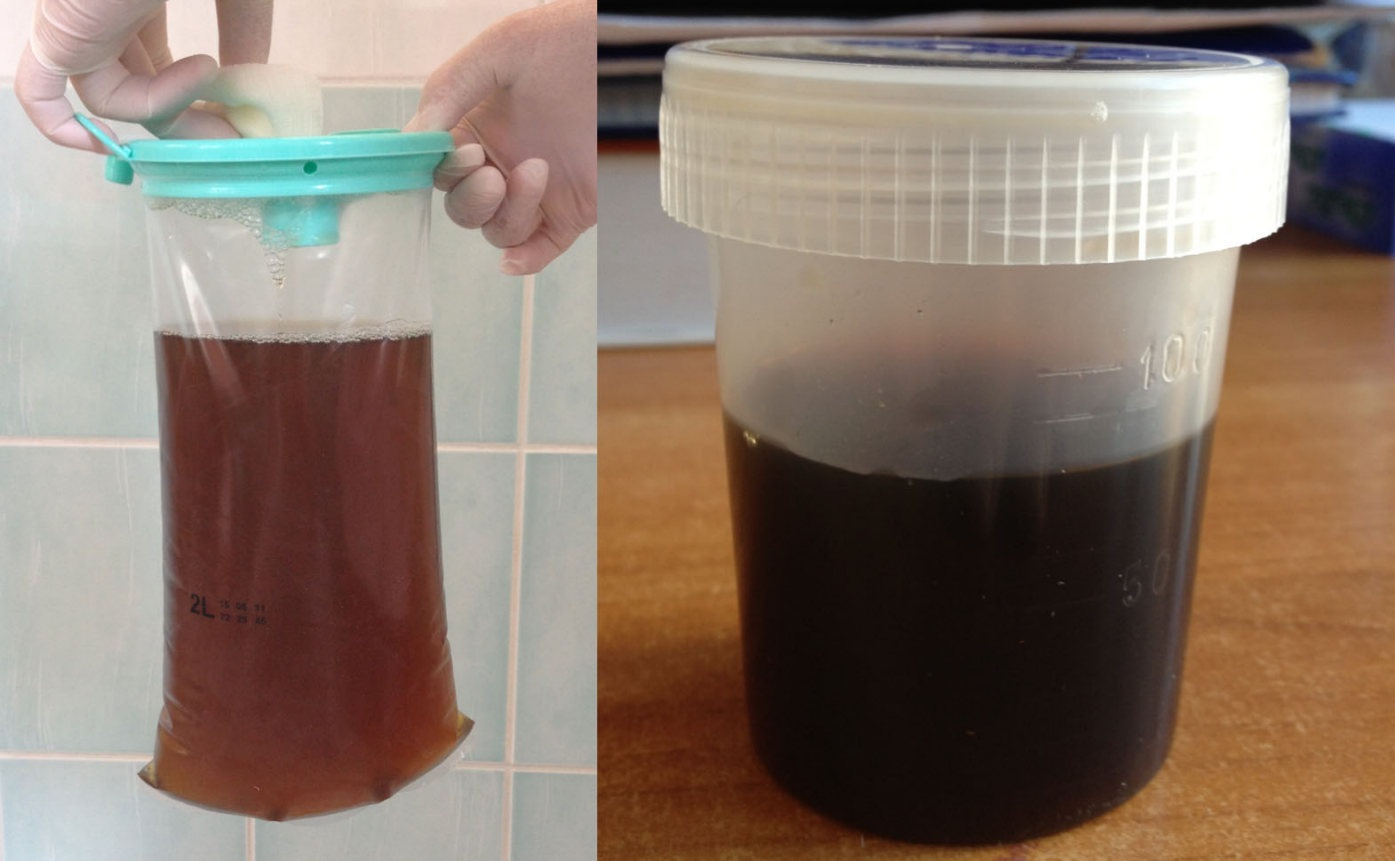
Dark urine (melanuria).

However, histopathological examination did not confirm the presence of cancer cells either in the surgical scar or in the removed lymph nodes. Subsequently, the patient was closely monitored for the development of generalized melanosis, which became apparent after undergoing angioplasty for unstable angina. The darkening of the skin, primarily affecting the face and torso (**Figure 1A**), began to manifest rapidly (**Figure 1B**). following the cardiac procedure. Initially, this skin discoloration and melanuria (darkened urine) were unaccompanied by other symptoms, and imaging studies failed to reveal signs of metastasis (**Figure 2**). Laboratory tests in the early stages of the disease revealed leukocytosis (13.4 × 10^3/µL), elevated cholesterol levels (227.89 mg/dL), and an increased erythrocyte sedimentation rate (36 mm/h).

After approximately four months, the patient began to experience weakness, though no significant weight loss occurred, and abdominal distension became noticeable. The patient was then referred to the internal medicine department, where laboratory tests revealed mild polycythemia (HGB 19.3 g/dL), hypercholesterolemia (268 mg/dL), elevated LDH (506 U/L), heightened ALP (alkaline phosphatase) (134 U/L), increased levels of Aspartate aminotransferase (Aspat) (65 U/L), Alanine aminotransferase (Alat) (42 U/L), total bilirubin elevation (1.6 mg/dL), increased indirect bilirubin (1.3 mg/dL), and elevated direct bilirubin (0.3 mg/dL). C-reactive protein (CRP) was also elevated (27.3 mg/L), while albumin levels were decreased (3.1 g/dL). Notably, urinalysis did not reveal significant abnormalities, except for the dark color of the urine.

**Figure 3:**
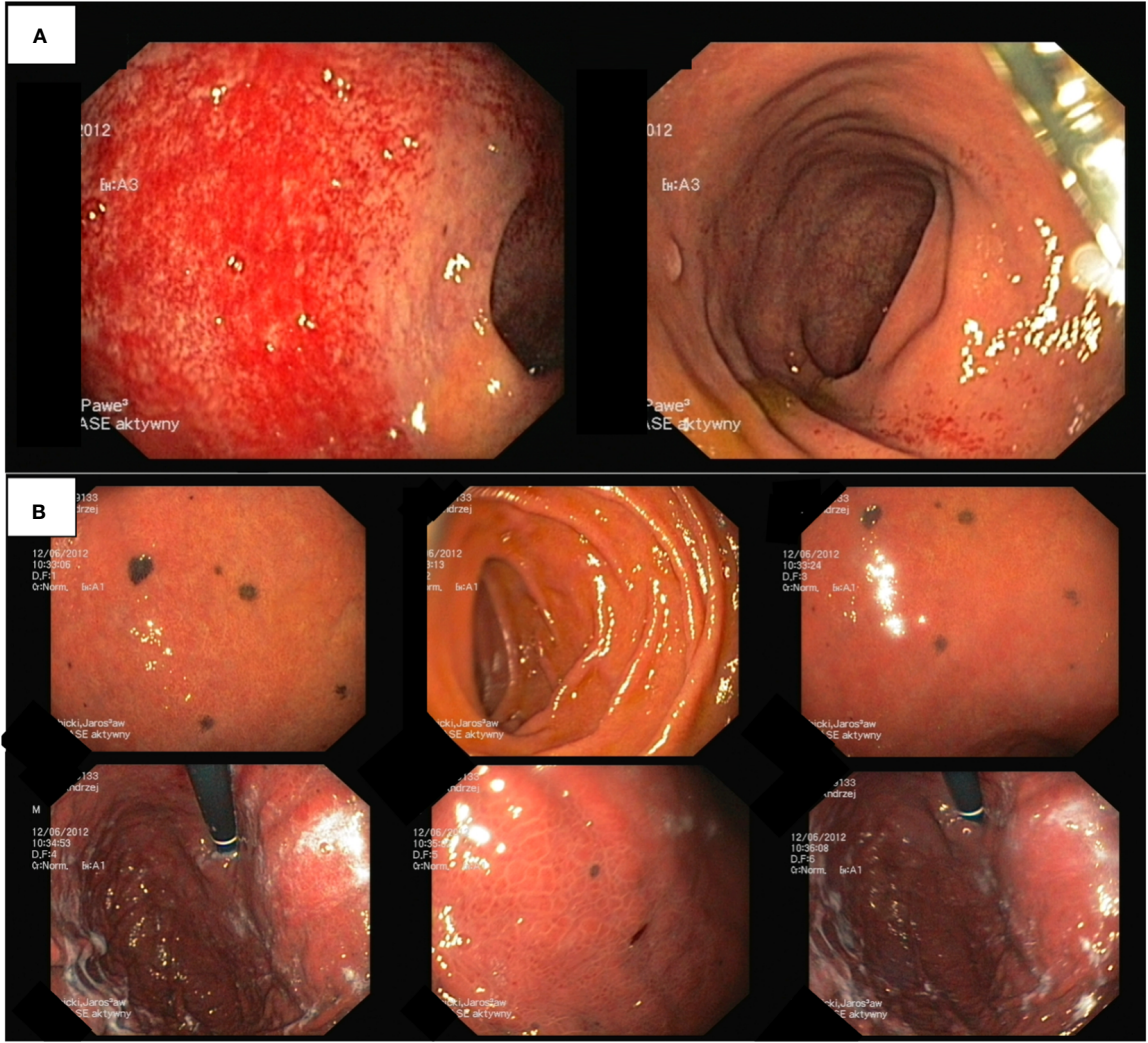
**(A)** Colonoscopy findings include colonic mucosa edema, mild inflammation, and small polyps. **(B)** Endoscopy of the upper gastrointestinal tract.

Given the absence of a definitive diagnosis and ongoing health concerns, further diagnostic evaluations were initiated. Abdominal ultrasound unveiled a tumor in the right kidney, measuring approximately 3.5 cm. Neck ultrasound identified enlarged lymph nodes, each up to 15 mm in size, without substantial structural abnormalities. Thoracic CT scan displayed enlarged subcarinal lymph nodes, each measuring up to 15 mm, without signs of clustering or evident pathological structure. Colonoscopy did not detect significant abnormalities, except for edema of the colonic mucosa, mild inflammation with minor contact bleeding, and a few small polyps that were removed during the examination (**Figure 3A**). Endoscopy of the upper gastrointestinal tract revealed dark discoloration of the duodenal bulb, accompanied by pigment spots up to 3 mm in size, with no apparent pathological findings (**Figure 3B**). According to the endoscopist, this unusual presentation could suggest metastasis to the gastric mucosa. Nevertheless, histopathological examination failed to confirm the presence of malignancy.

Positron Emission Tomography (PET) scan revealed inconclusive findings in the liver and right kidney. Assessment of the bladder proved challenging due to highly radioactive urine. Additionally, laboratory tests indicated a gradual increase in hemoglobin levels, leukocytosis, elevated CRP, and heightened LDH.

In light of unexplained generalized melanosis, a thorough dermatoscopic examination of the entire skin was conducted, revealing a darker pigmentation with a gray-blue hue on the discolored skin of the left arm. The dermatoscopic image suggested a metastatic change in the deep layers of the skin. A decision was made to proceed with further diagnostics at the Dermatology Department. A surgical biopsy of the skin on the left arm ultimately confirmed the diagnosis of melanoma metastasis. Additionally, an independent investigation of BRAF mutations was carried out, confirming a pathological alteration in codon V600E During the patient’s stay in the Dermatology Department, LDH levels continued to rise (596 U/L), and inflammatory markers remained elevated. Notably, while all tumor markers, including CEA, Ca 15.3, Ca 19.9, and PSA, were within normal ranges, S100 protein levels were significantly elevated (19.32 µg/L).

The patient was subsequently transferred to the Lower Silesian Oncology Center with a confirmed diagnosis of metastatic melanoma. Hemoglobin levels continued to rise (19.6 g/dL), and various other laboratory values, including calcium, uric acid, albumin, bilirubin, transaminases, ALP, GGTP, and LDH, were found to be abnormal. A hematologist’s consultation attributed the erythrocytosis to the underlying disease, prompting a decision to forego a bone marrow biopsy. Bloodletting was performed, and the patient was prescribed allopurinol, low-dose aspirin, and an increased fluid intake.

On July 25, 2012, the patient commenced the first cycle of DTIC (dacarbazine) chemotherapy, with moderate tolerance, albeit accompanied by side effects such as nausea, diarrhea, and increasing weakness. In Poland at that time there was no reimbursement access to BRAF and MEK inhibitors as well as immunotherapy. During the course of treatment, abdominal distension was observed due to ascites. A paracentesis was conducted, yielding approximately 3 liters of amber fluid. S100 protein and other markers continued to rise.

**Figure 4:**
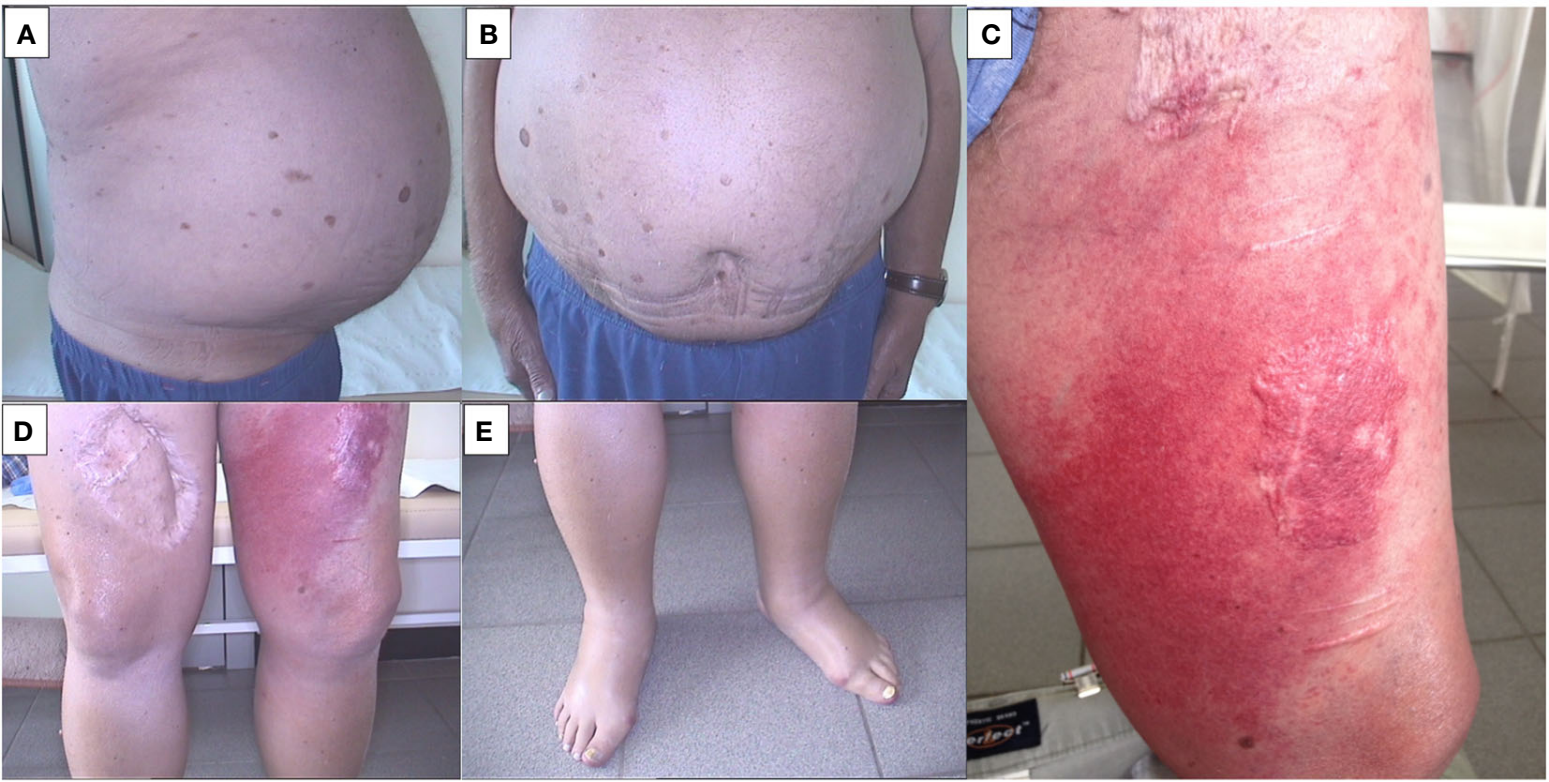
**(A)** Ascites (side photo).; **(B)** Ascites (front photo).; **(C)** Rapidly progressing cellulitis (erysipelas).; **(D)** Pedal edema.; **(E)** Rapidly progressing cellulitis (erysipelas).

Following the completion of the first chemotherapy cycle on July 29, 2012, the patient’s weakness progressively worsened. On August 1, 2012, the patient presented with localized erythema, swelling, and increased warmth in the lower left extremity, indicative of rapidly progressing cellulitis (erysipelas) (**Figures 4A–E**).

Antibiotics, antipyretics, anti-inflammatory medications, antifungals, and low molecular weight heparin were initiated, coupled with the elevation of the affected limb and increased fluid intake. The subsequent day witnessed the onset of quantitative disturbances in the patient’s level of consciousness and a drop in blood pressure, culminating in their transfer to the hospital’s Intensive Care Unit. Regrettably, the patient passed away on August 3, 2012.

## Discussion

The role of melanogenesis in melanoma progression is complex and multifaceted. Melanin, produced through the process of melanogenesis, traditionally serves a protective function against the harmful effects of ultraviolet (UV) radiation and other environmental stressors (13). It is generated through the enzymatic conversion of L-tyrosine to dopaquinone, leading to the formation of various melanin oligomers, including constituents of eumelanin and pheomelanin (14–18). While melanin is known to provide protection against skin cancers, including cutaneous melanoma, its presence may also be implicated in the malignant transformation of melanocytes (19). The biosynthesis of melanin is regulated by various factors, including sun exposure and hormonal influences at different levels (20, 21). Importantly, the melanin pigment has been associated with both protective and potentially detrimental effects in the context of melanoma development (13). Eumelanin, considered a protective melanin type, acts as an efficient antioxidant and sunscreen, providing radioprotection and photoprotection (22). On the other hand, pheomelanin, less photostable, can create a mutagenic environment after exposure to short-wavelength UV radiation. Melanogenesis and its intermediates exhibit cytotoxic, genotoxic, and mutagenic activities. They can stimulate glycolysis and activate hypoxia-inducible factor 1-alpha (HIF-1α), contributing to melanoma progression and resistance to immunotherapy (23). Additionally, melanin pigments, particularly eumelanin, consume oxygen and can alter cellular metabolism, promoting aerobic glycolysis in melanotic melanomas. The stimulation of melanogenesis has been associated with changes in glycoproteins’ phosphorylation patterns and the upregulation of HIF-1-dependent and independent pathways. Paradoxically, while melanogenesis may contribute to melanoma progression, it also has inhibitory effects. Melanin can inhibit the formation of melanoma metastases in certain contexts. The intricate interplay between pro-oxidative conditions induced by melanogenesis and the inhibitory effects of melanin granules, due to their mechanical properties, further complicates the understanding of melanoma progression. Clinicopathological analyses have revealed correlations between melanization levels and the survival of cutaneous melanoma patients (23). Higher pigmentation in metastatic tumors has been linked to poorer prognosis, emphasizing the need to consider melanin content in therapeutic strategies (24). Accordingly, melanogenesis demonstrates a dualistic role in the development, progression, and treatment of melanoma.While melanin provides protection against UV-induced damage, its complex interactions within melanoma cells contribute to the disease’s progression. Understanding these intricacies is crucial for developing targeted therapeutic approaches and improving patient outcomes.

Generalized melanosis, is characterized by a rapid and aggressive progression, initially presenting as widespread melanosis covering the entire body, with pronounced pigmentation on the face, neck, and distal extremities (areas more exposed to sunlight) (9, 25–27). The initially subtle change in color, often described as a slate blue or even cerulean blue shade, tends to go unnoticed in many instances of widely spread metastatic melanoma (28). This early bluish hue is a sign that the melanin buildup is starting in the deeper layers of the skin. In the rare cases where patients survive long enough to experience the full development of melanosis, the pigment accumulation advances swiftly, resulting in a very dark skin tone by the time of their demise. From a histological perspective, the majority of documented cases commonly exhibit the existence of dermal pigment, the accumulation of melanin within histiocytes, and the presence of unbound melanin in the dermal connective tissue (28, 29). One consistent discovery in generalized melanosis involves the accumulation of melanin pigment in the deeper layers of the dermis, primarily concentrated within macrophages situated near small blood vessels (8). Studies conducted on diffuse cutaneous melanosis indicate mean time to mortality of around 4 to 5 months following the onset of the disease. Managing generalized malignant melanoma poses a significant challenge in the realm of oncology, primarily relying on palliative measures. To explore viable treatment options, there is an ongoing imperative for persistent and methodical research efforts (30, 31). Little is known about genetic mutations in generalized melanoma. However, some studies revealed BRAF mutations in patients with melanuria (32).

The disease typically follows a fulminant course and is often refractory to conventional chemotherapy. The patient under consideration harbored a BRAF mutation in the primary lesion. Due to formal reasons, treatment with BRAF inhibitors could not be initiated. Consequently, the patient commenced chemotherapy with DTIC (Dacarbazine), which unfortunately resulted in rapid disease progression, culminating in the patient’s demise. This case underscores that patients with generalized melanosis experience a more accelerated disease progression than usual and are not responsive to conventional chemotherapeutic treatments. It appears that this patient profile necessitates the exploration of rapidly acting, molecularly targeted therapies, such as BRAF or C-KIT inhibitors, in alignment with the genetic profile, rather than immunotherapy. However, it is worth noting that melanomas in advanced stages possess a remarkable ability to produce various classical endocrine hormones, such as pituitary and hypothalamic hormones, enkephalins, catecholamines, and corticosteroids (33–35). This diverse secretion profile allows melanomas to intricately influence the body’s neuroendocrine centers, manipulating homeostasis to create a favorable environment for tumor expansion and concurrently establishing resistance against immunotherapeutic interventions.

The challenges encountered in diagnosing and managing generalized melanosis and associated metastatic melanoma are underscored in this case. Early detection, effective interventions, and advancements in understanding the disease mechanisms remain critical for improving patient outcomes. The swift progression and diverse clinical manifestations witnessed in this case emphasize the intricacies involved in managing advanced melanoma and the necessity for more efficacious treatment modalities. Furthermore, since changes in the amount of cellular eumelanin and pheomelanin have been linked to carcinogenesis, measuring the relative concentrations of these compounds may prove to be an interesting scientific procedure (36). Consequently, further research is warranted to enhance our comprehension of melanuria and to develop targeted therapeutic approaches for patients grappling with metastatic melanoma and its associated complexities. Will the swift implementation of targeted therapy yield therapeutic effects, and will the regression of the lesions also lead to the disappearance of melanosis in the skin, mucous membranes, and conjunctiva? Is it coincidental that a skin biopsy from the highly pigmented area revealed melanoma and not just skin cells rich in melanin? We are still awaiting answers to many of these and other questions. Descriptions of patients with fulminant progression of generalized melanosis may influence the therapeutic decisions of physicians who encounter similar cases in the future similar cases.

## Conclusions

This case highlights the challenges in diagnosing and managing generalized melanosis and its metastatic melanoma. Early detection and more effective treatments are crucial. Further research is needed to understand this process. Questions remain regarding the efficacy of targeted therapy in reducing melanosis and the significance of melanoma in highly pigmented skin areas. This case provides insights for future similar cases, influencing physicians’ therapeutic decisions.

## Data availability statement

The raw data supporting the conclusions of this article will be made available by the authors, without undue reservation.

## Ethics statement

Written informed consent was obtained from the individual(s) for the publication of any potentially identifiable images or data included in this article.

## Author contributions

JC: Conceptualization, Investigation, Project administration, Supervision, Visualization, Writing – original draft, Writing – review & editing. PD: Formal analysis, Funding acquisition, Project administration, Supervision, Writing – review & editing. NS: Conceptualization, Formal Analysis, Funding acquisition, Visualization, Writing – original draft, Writing – review & editing.
